# Combining different points of view on plant descriptions: mapping agricultural plant roles and biological taxa

**DOI:** 10.3389/frai.2023.1188036

**Published:** 2023-09-27

**Authors:** Florence Amardeilh, Sophie Aubin, Stephan Bernard, Sonia Bravo, Robert Bossy, Catherine Faron, Franck Michel, Juliette Raphel, Catherine Roussey

**Affiliations:** ^1^Elzeard, Bordeaux, France; ^2^DipSO, INRAE, Paris, France; ^3^Université Clermont Auvergne, INRAE, UR TSCF, Clermont-Ferrand, France; ^4^MaIAGE, INRAE, Université Paris-Saclay, Jouy-en-Josas, France; ^5^Université Côte d'Azur, Inria, I3S, Sophia-Antipolis, France; ^6^MISTEA, University of Montpellier, INRAE & Institut Agro, Montpellier, France

**Keywords:** mapping, SSSOM, crop, taxa, thesaurus French Crop Usage, French national taxonomic repository, TAXREF, SKOS model

## Abstract

This article describes our study on the alignment of two complementary knowledge graphs useful in agriculture: the thesaurus of cultivated plants in France named French Crop Usage (FCU) and the French national taxonomic repository TAXREF for fauna, flora, and fungi. FCU describes the usages of plants in agriculture: “*tomatoes*” are crops used for human food, and “*grapevines*” are crops used for human beverage. TAXREF describes biological taxa and associated scientific names: for example, a tomato species may be “*Solanum lycopersicum*” or a grapevine species may be “*Vitis vinifera*”. Both knowledge graphs contain vernacular names of plants but those names are ambiguous. Thus, a group of agricultural experts produced some mappings from FCU crops to TAXREF taxa. Moreover, new RDF properties have been defined to declare those new types of mapping relations between plant descriptions. The metadata for the mappings and the mapping set are encoded with the Simple Standard for Sharing Ontological Mappings (SSSOM), a new model which, among other qualities, offers means to report on provenance of particular interest for this study. The produced mappings are available for download in Recherche Data Gouv, the federated national platform for research data in France.

## 1. Introduction

While the Web of linked data makes more and more knowledge graphs available, their cross-use often remains a challenge. This study presents a dataset containing mappings between two knowledge graphs representing different points of view on the same objects. This mapping set should allow to query simultaneously these graphs to enrich object descriptions by combining these points of view. Agriculture offers a particular use case of mappings, linked to the modeling of cultivated plants. Several expertises are needed to describe a cultivated plant: farmer vs. agronomist, agronomist vs. ecologist. The scientific world (ecologists or agronomists) tends to use scientific names from taxonomic science to designate living organisms (plants, insects). These scientific names are stored in biological taxonomies. The world of users (farmers) generally uses vernacular names or domain specific categories (e.g., cereals) to designate the living organisms involved in their practice. In parallel, a plant can have several usages in agriculture: (1) plants cultivated in a plot for production purposes such as vegetables or cereals, in other word crops, (2) weeds that appear on a plot without being cultivated for which farmers want to limit the development or remove them from the plot, (3) a first cultivated plant that provides a service to a second cultivated plant for production purposes. Both plants are cultivated on the same plot but sometime not at the same time. The first cultivated plant will be destroyed without being harvested and is called service plant. The second cultivated plant will be harvested and is called the crop. For reasons of conciseness, we will limit this article to the plant usages for production purposes that is to say crops.

We present our study on the mappings of two complementary knowledge graphs useful in the agricultural domain: the French Crop Usage thesaurus (FCU) and the French national taxonomic register TAXREF for fauna, flora, and fungi. FCU describes the usage of plants in agriculture: “*tomatoes*” are crops used for human food, “*grapevines*” are crops used for human food or beverage. It represents the farmers' point of view. TAXREF describes biological taxa and associated scientific names: for example, a tomato species may be “*Solanum lycopersicum*” or a grapevine species may be “*Vitis vinifera*”. TAXREF represents the agronomists' point of view. Both knowledge graphs contain vernacular names of plants. Vernacular names are often ambiguous and not consensual, which renders the matching activity particularly challenging.

Our previous studies (Michel et al., 2022) have implemented several automatic alignment methods based on vernacular names comparison. Those automatic methods reused existing reference sources such as EPPO global database^1^ and the official French catalog of species and varieties of cultivated plants GEVES.^2^ The results show that it is necessary to clean the automatically produced alignments due to the ambiguity of vernacular names. Therefore, a group of agricultural experts has produced a set of valid mappings. Those mappings are published as open data on the French Recherche Data Gouv repository.^3^ Thus, they could be used as a gold standard to validate any automatic alignment methods.

The remainder of the study is organized as follows: Section 2 describes first the knowledge graphs and vocabularies used in our mapping set (Section 2.1), followed by the manual method applied to align the two knowledge graphs (Section 2.2). Section 3 presents our analysis of the challenge encountered in matching the graphs and representing the mapping set using the SSSOM model. In Section 4, we summarize the results and provide an outlook for future improvement.

## 2. Methods

### 2.1. Materials

First, we describe in detail the two aligned knowledge graphs: TAXREF-LD and FCU. Second we present the RDF vocabulary that we defined to declare new types of mapping relations between plant descriptions. Indeed, SKOS properties are not sufficient to align an agricultural usage with a scientific taxon. Third, the SSSOM vocabulary is presented to store the set of mappings and their metadata.

#### 2.1.1. TAXREF and TAXREF-LD

TAXREF (Gargominy et al., 2021) is the French taxonomic repository for fauna, flora, and fungi. In addition to a Web portal, a REST service, and a set of downloadable CSV files, TAXREF is available in the form of a knowledge graph complying with the Linked Data principles, named TAXREF-LD (Michel et al., 2017). TAXREF-LD is available on the AgroPortal repository.^4^ This study has been developed using the 15.2 version of TAXREF-LD which contains 287,229 classes and more than 1,000,000 instances.

**Figure 1:**
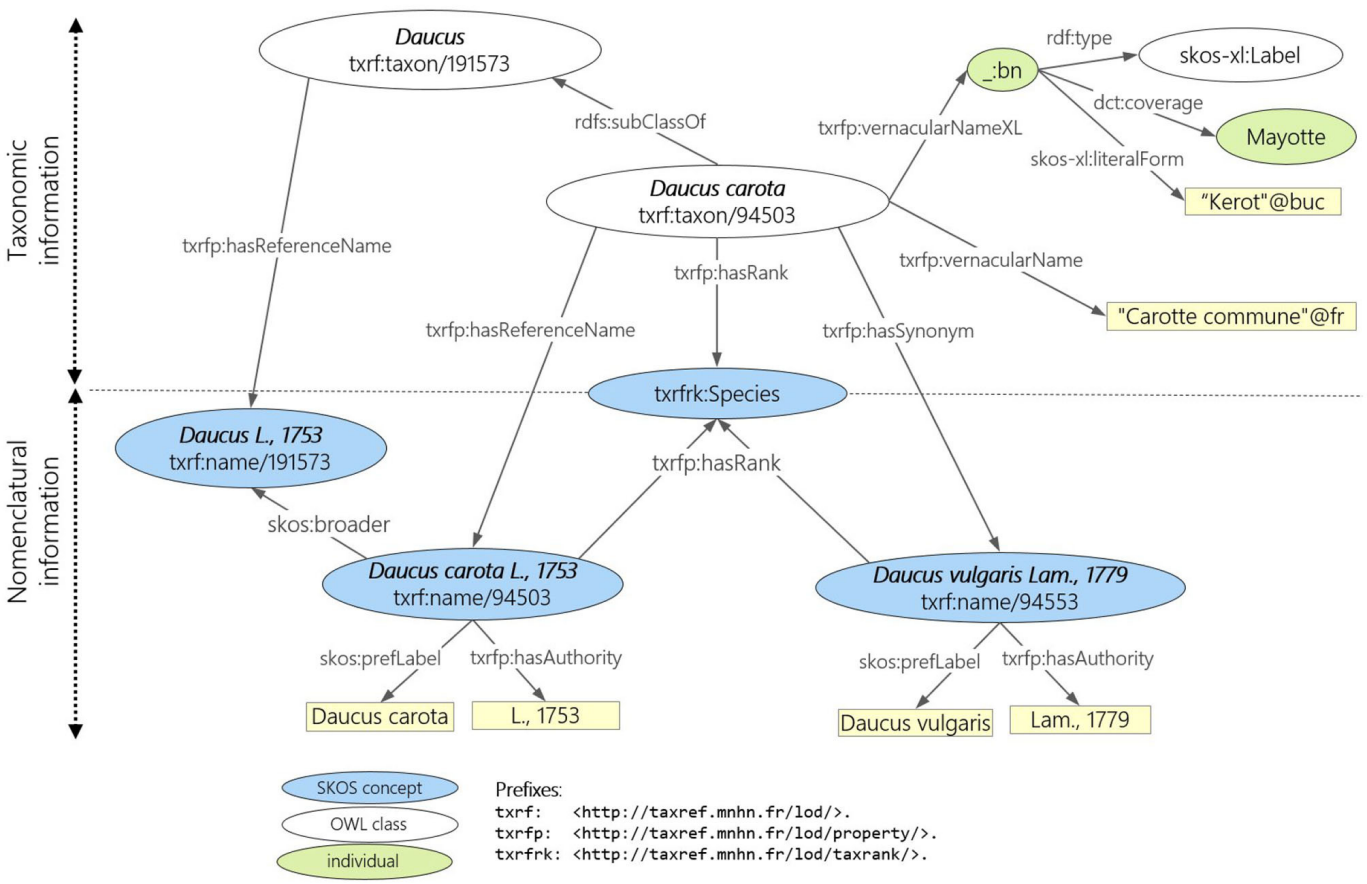
Modeling principle of TAXREF-LD distinguishing taxonomic and nomenclatural information. The species taxon “*Daucus carota*” has for scientific name “*Daucus carota* L, 1758”.

To accurately reflect the distinction between taxonomy (a taxon gathers biological individuals that share common characteristics) and nomenclature (the scientific names assigned to taxa), TAXREF-LD has two distinct levels of modeling, as shown in Figure 1. At the taxonomic level, each taxon is modeled as an OWL class whose members are the biological individuals of that taxon. The parent class is the higher ranked taxon (e.g., “*Daucus carota*” is of rank species, the parent class “*Daucus*” is of rank genus). At the nomenclatural level, scientific names are represented as concepts in a SKOS thesaurus. Each name (instance of *skos:Concept*) is linked to a taxon (an *OWL class*) by a property indicating whether it is the reference name (*accepted name* in zoology or *valid name* in botany) or a synonym. The figure also presents vernacular names that are represented as a simple literal as well as a blank node of type *skos-xl:Label* that reifies the vernacular name and makes it possible to provide additional information such as the geographic area in which this vernacular name is valid or a bibliographic reference. In addition to strictly taxonomic information, TAXREF-LD also represents other types of information not shown on this figure, such as habitats, conservation status, biogeographical status, interactions between species, and the bibliographical references associated with this information. Notably, TAXREF-LD sometimes associates the same vernacular name with several taxa. These vernacular names are taken from the publications where the scientific names are declared. Furthermore, TAXREF-LD is linked to several third-party taxonomic repositories including Agrovoc Thesaurus and NCBI Organismal Taxonomy.

#### 2.1.2. French Crop Usage thesaurus

The French Crop Usage (FCU) thesaurus normalizes crop names in French. Moreover, it organizes these crop names in categories, according to their usages on the French territory. The usages represent also the agricultural sectors.

**Figure 2:**
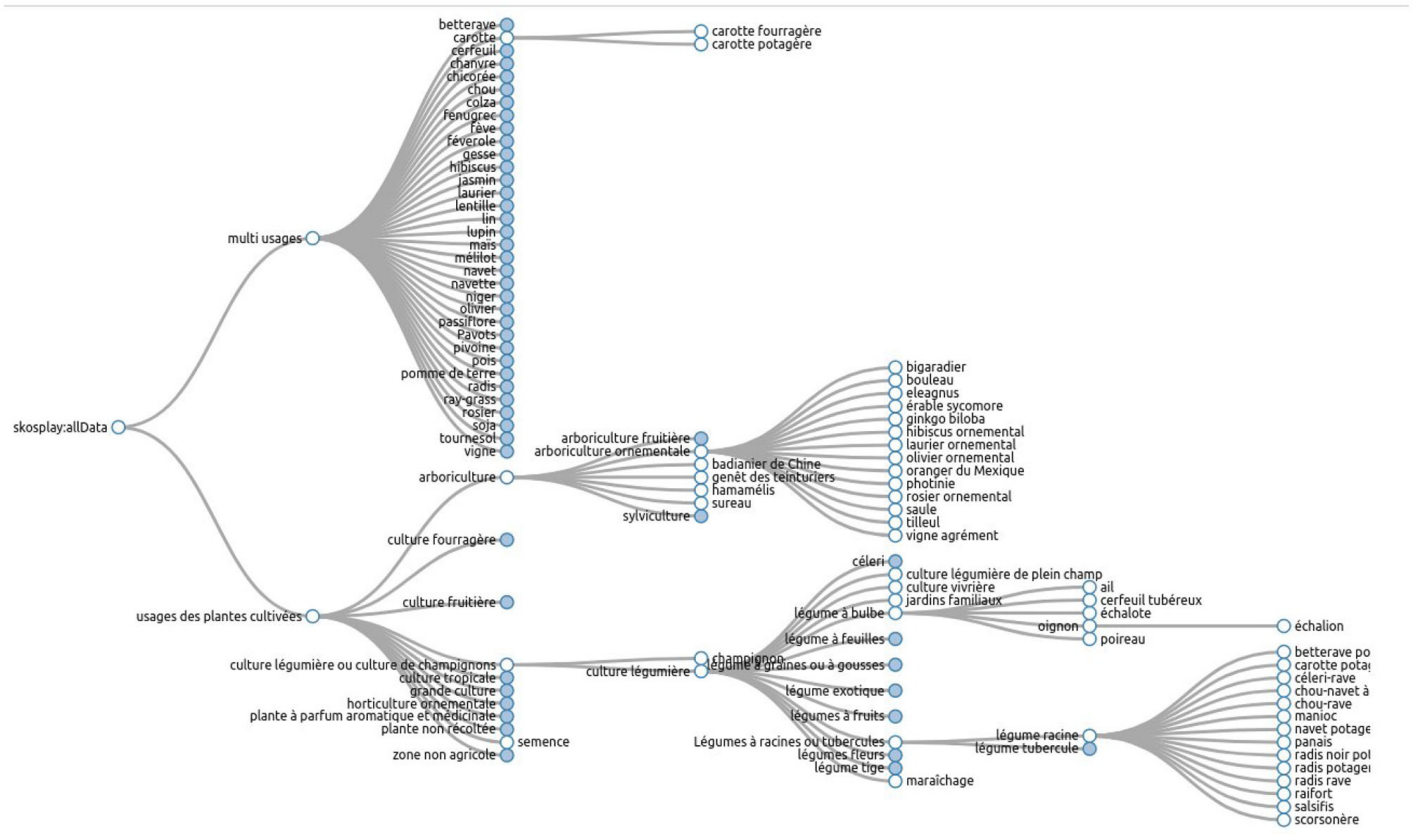
An extract from the FCU thesaurus, visualized with the SKOS Play tool.

As shown in Figure 2, the thesaurus hierarchy has two main branches. The branch named “*Multiusages*” contains all the cultivated plants that have several usages in agriculture. For example, “*carotte*” (carrot) may be used as vegetable or fodder. The branch “*Usages*_*plantes*_*cultivees*” organizes cultivated plants according to their usages and represents agricultural sectors. In this branch, the crop usage “*carotte potagère*” is linked to the vegetable category “*légume racine*” (root vegetable).

The FCU thesaurus is formalized using the Simple Knowledge Organization System (SKOS) vocabulary proposed by W3C (Miles and Bechhofer, 2009). Each crop usage or category is represented by an instance of *skos:Concept*. The thesaurus is published on the Web using Linked Data principles.

**Figure 3:**
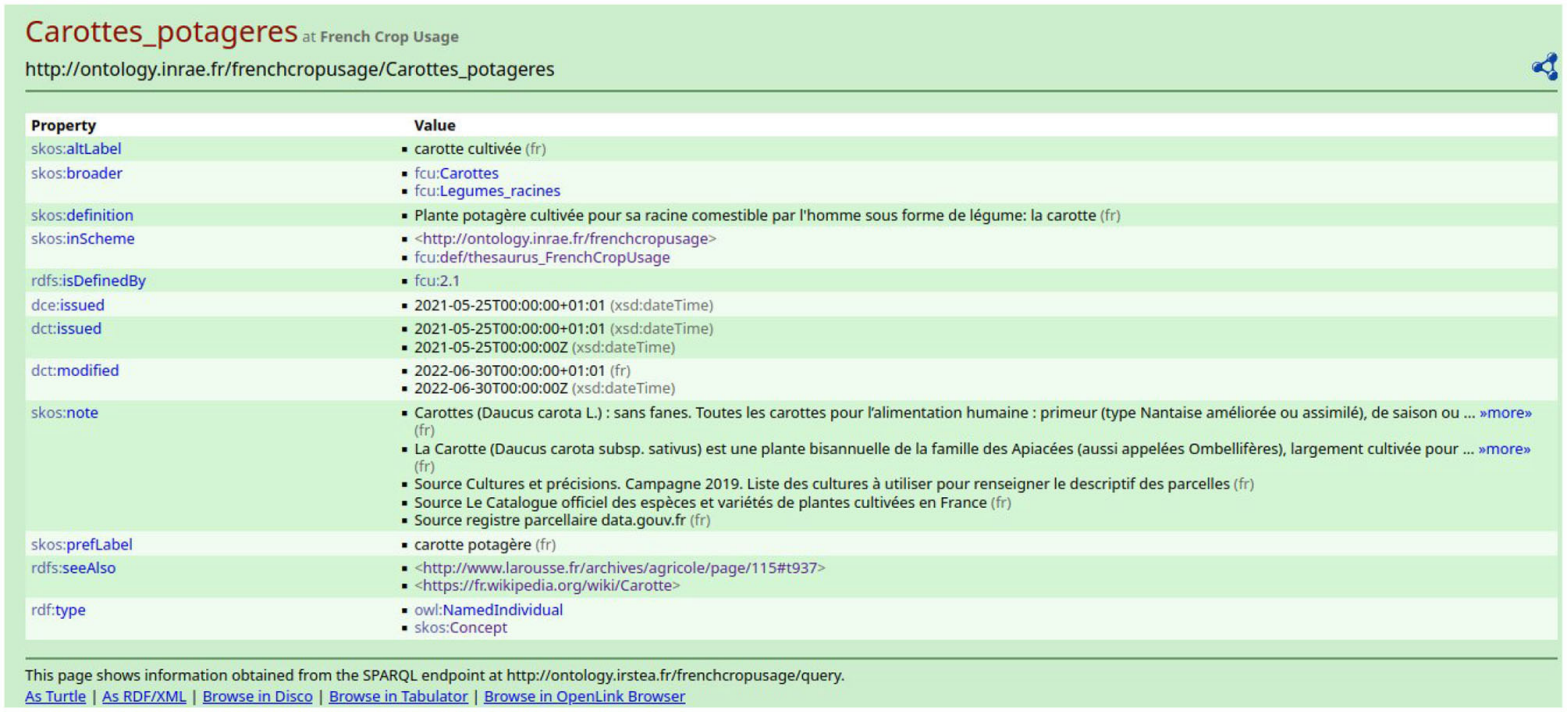
The information related to the *skos:Concept* instance “*fcu:Carottes_potageres*”.

The thesaurus is available on the AgroPortal repository.^5^ This study has been developed using the 3.3 version of FCU which contains 707 instances of *skos:Concept*. The maximum depth of the hierarchy is 6 levels. Each *skos:Concept* is defined by several properties, as shown in Figure 3. The description of a crop usage or category contains the following:

- The value of property *skos:prefLabel* is the crop name in French. The term is the vernacular name of the cultivated plant or the category name. To avoid ambiguity in the case of a cultivated plant with different usages, the crop name is the combination of the vernacular name of the plant and its usage. For example, in Figure 3, the crop name is “*carotte*” + “*potagère*”.
- The value of property *skos:altLabel* is other possible labels that can be used for the crop. For example, in Figure 3, an alternative crop name is “*carotte cultivée*”.
- The value of property *skos:definition* is the definition of the crop usage in French. The definition accounts for the crop position in the hierarchy.
- The value of property *skos:note* is at least one definition from another source, such as the French Wikipedia. The definition always ends by the indication of the source. For example, in Figure 3, the crop “*carotte potagère*” was found in The *Official Catalog of Species and Varieties of Cultivated Crops in France*.^6^ Thus, depending on the source, the same crop may have different names which show the ambiguity of crop names.

#### 2.1.3. Mapping properties: taxon vs. usage

In TAXREF-LD, a taxon is defined by an *OWL class*, and the names of taxon are defined by instances of *skos:Concept*. In FCU, a crop usage is defined by an instance of *skos:Concept*.

We have defined 10 annotation properties to link an *OWL class* representing a taxon to an instance of *skos:Concept* representing a crop usage. The main annotation property is *ontofcu:hasTaxon* (/ its inverse property is *ontofcu:hasUsage*). This property (/ its inverse property) links a crop usage to a taxon. This relation indicates that the taxon is a candidate to fulfill the crop usage. For example, the species “*Daucus Carota*” can be used as “*carotte fourragère*” (fodder carrot). This property is specialized into four annotation properties (and their inverse) as follows:

- *ontofcu:hasGenericTaxon* (/ *ontofcu:hasSpecificUsage*) annotation property represents a relationship from a crop usage to a taxon. This relation indicates that one of the descendants of the taxon is the reference taxon to fulfill the crop usage. It is used when the descendant is not defined in the taxonomy source (e.g., TAXREF-LD). For example, the form “*Cichorium intybus var. foliosum Hegi f. cylindricum*” is known to fulfill the vegetable usage “*chicorée pain de sucre*” (sugarloaf chicory). Unfortunately, this form does not appear in TAXREF-LD. Its parent, the variety “*Cichorium intybus var. Foliosum*,” belongs to TAXREF-LD. Thus, the mapping between “*chicorée pain de sucre*” (sugarloaf chicory) and the variety “*Cichorium intybus var. Foliosum*” will use the property *ontofcu:hasGenericTaxon*.
- *ontofcu:hasInvalidTaxon* (/ *ontofcu:hasInvalidUsage*) annotation property represents a relationship from a crop usage to a taxon. This relation indicates that the taxon can not be used to fulfill the crop usage. For example, the subspecies “*Daucus carota subsp. gadecaei*” is not a cultivated plant and can not be used as “*carotte potagère*” (vegetable carrot). This property is used to invalidate the output of automatic alignment tool.
- *ontofcu:hasReferenceTaxon* (/ *ontofcu:hasReferenceUsage*) annotation property represents a relationship from a crop usage to a taxon. This relation indicates that the taxon is the reference known to fulfill the crop usage. For example, the species “*Daucus Carota*” is one of the reference taxa used as “*carotte potagère*” (vegetable carrot). The subspecies “*Daucus carota subsp. sativus*” is another reference taxon to be used as “*carotte potagère*” (vegetable carrot).
- *ontofcu:hasSpecificTaxon* (/ *ontofcu:hasGenericUsage*) annotation property represents a relationship from a crop usage to a taxon. This relation indicates that one of the descendants of the crop usage is the reference usage of the taxon. It is used when the descendant of the crop usage is not defined in FCU. For example, the variety “*Solanum lycopersicum var. cerasiforme*” is known to fulfill the crop usage “*tomate cerise*” (cherry tomato). Unfortunately, this type of tomato is not defined in FCU. Thus, the mapping between “*tomate*” (tomato) and the variety “*Solanum lycopersicum var. cerasiforme*” will use the property *ontofcu:hasSpecificTaxon*.

We have defined 12 object properties to link an instance of *skos:Concept* representing a scientific name to an instance of *skos:Concept* representing a crop usage. Those object property triples should be associated with annotation property triples listed above used as documentation. The main object property is *ontofcu:hasScientificName* (/ *ontofcu:hasVernacularName*). This property (/ its inverse property) is a relation from a crop usage to a taxon scientific name. Both are represented as an instance of *skos:Concept*. This relation indicates that the taxon scientific name is a candidate to identify the crop usage. For example, “*Daucus carota L., 1753*” can be the scientific name of the crop usage “*carotte fourragère*” (fodder carrot). Notably, the crop usage and the associated taxon should also be linked by the annotation property *ontofcu:hasTaxon* / *ontofcu:hasUsage*.

- *ontofcu:hasGenericScientificName* (/ *ontofcu:hasSpecificVernacularName*) object property represents a relationship from a crop usage to a taxon scientific name. This relation indicates that one of the descendants of the taxon name is known to be the scientific name of the crop usage. This is used when the descendant is not defined in the taxonomy. For example, the form scientific name “*Cichorium intybus var. foliosum Hegi f. cylindricum*” is known to be the scientific name of the vegetable usage “*chicorée pain de sucre*” (sugarloaf chicory). Unfortunately, this form does not belong to TAXREF-LD. But its parent, the variety scientific name “*Cichorium intybus var. Foliosum Hegi, 1928*” belongs to TAXREF-LD. Thus, the mapping between “*chicorée pain de sucre*” (sugarloaf chicory) and the variety scientific name “*Cichorium intybus var. Foliosum Hegi, 1928*” will use the property *ontofcu:hasGenericScientificName*. Notably, the crop usage and the associated taxon should also be linked by the annotation property *ontofcu:hasGenericTaxon*.
- *ontofcu:hasInvalidScientificName* (/ *hasInvalidVernacularName*) object property represents a relationship from a crop usage to a taxon scientific name. This relation indicates that the taxon scientific name can not be used to identify the crop usage. For example, the subspecies “*Daucus carota subsp. gadecaei*” is not a cultivated plant. Thus, “*Daucus carota subsp. gadecaei (Rouy & E.G.Camus) Heywood, 1968*” is not the scientific name of “*carotte potagère*” (vegetable carrot). Notably, the crop usage and the associated taxon should also be linked by the annotation property *ontofcu:hasInvalidTaxon*.
- *ontofcu:hasReferenceScientificName* (/ *ontofcu:hasReferenceVernacularName*) object property represents a relationship from a crop usage to a taxon scientific name. This relation indicates that the taxon scientific name can be used to identify the crop usage. For example, “*Daucus carota L., 1753*” is the reference scientific name of “*carotte fourragère*” (fodder carrot). Notably, the crop usage and the associated taxon should also be linked by the annotation property *ontofcu:hasReferenceTaxon*.
- *ontofcu:hasSpecificScientificName* (/ *ontofcu:hasGenericVernacularName*) object property represents a relationship from a crop usage to a taxon scientific name. This relation indicates that one of the descendants of the crop usage is the reference vernacular name of the taxon. This is used when the descendant of the crop usage is not defined in FCU. For example, “*Solanum lycopersicum var. cerasiforme (Alef.) Fosberg, 1955*” is known to be the scientific name of the crop usage “*tomate cerise*” (cherry tomato). Unfortunately, “*tomate cerise*” does not belong to FCU. Thus, the mapping between “*tomate*” (tomato) and the variety scientific name “*Solanum lycopersicum var. cerasiforme (Alef.) Fosberg, 1955*” will use the property *ontofcu:hasSpecificScientificName*. Notably, the crop usage and the associated taxon should also be linked by the annotation property *ontofcu:hasSpecificTaxon*.
- *ontofcu:hasSynonymousScientificName* object property represents a relationship from a crop usage to a taxon scientific name. This relation indicates that the scientific name is not the reference name but one of its synonyms and can also be used to identify the crop usage. For example, “*Daucus communis Rouy & E.G.Camus, 1901*” is the synonymous scientific name of “*carotte fourragère*” (fodder carrot). Notably, the crop usage and the associated taxon should also be linked by the annotation property *ontofcu:hasReferenceTaxon*.
- *ontofcu:hasSynonymousVernacularName* object property represents a relationship from a taxon scientific name to a crop usage. This relation indicates that the crop usage is the synonymous vernacular name of the taxon scientific name. For example, FCU contains a new collection of crop usage dedicated to subsistence crops. A new crop usage “*carotte du jardin*” (garden carrot) is added which is defined as synonymous (same as) to “*carotte potagère*” (vegetable carrot). Thus, “*carotte du jardin*” (garden carrot) will be the synonymous vernacular name of “*Daucus carota L., 1753*.” Notably, the both crop usages and the associated taxon should also be linked by the annotation property *ontofcu:hasReferenceTaxon*.

**Figure 4:**
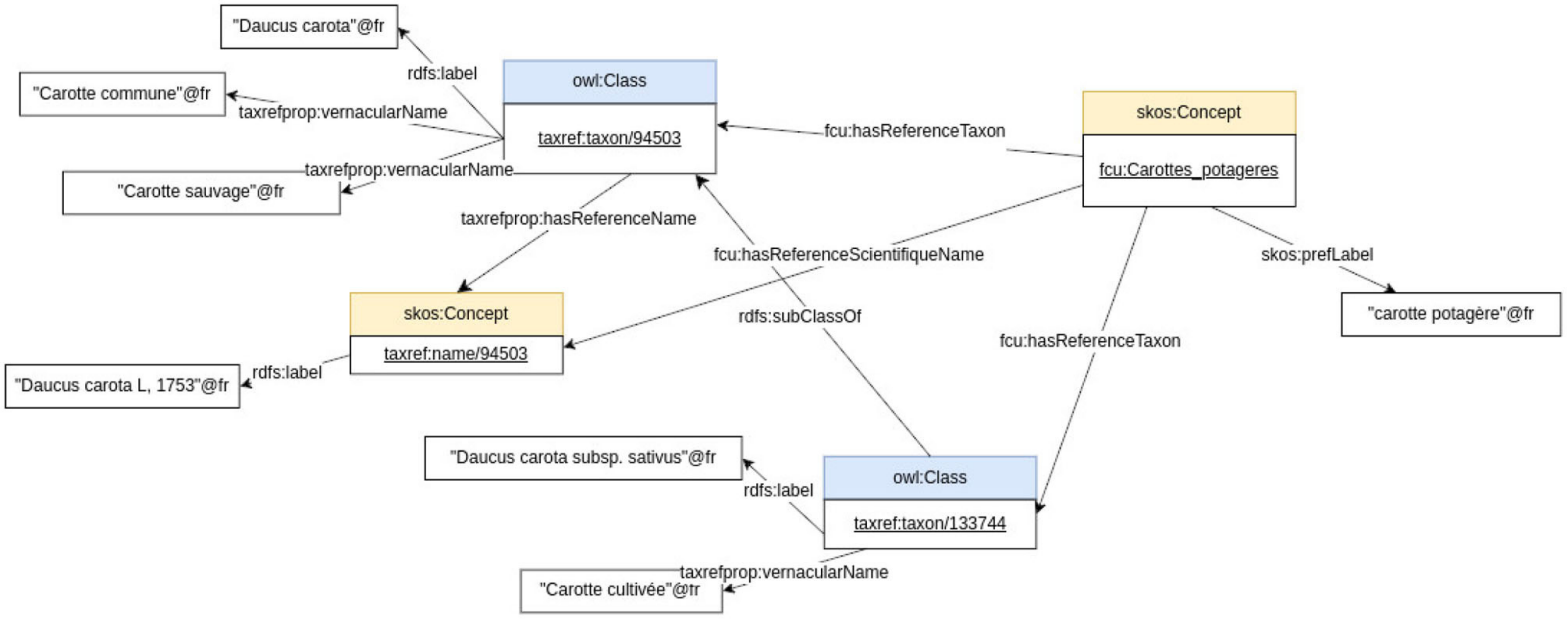
Some mappings between the crop usage “*carotte potagère*”, the taxon “*Daucus carota*”, its scientific name “*Daucus carota* L, 1758”, and the taxon “*Daucus carota* subsp. *sativus*” using the CHOWLK language.

Figure 4 shows an exception of the mapping between the FCU concept “*carotte potagère*” and two TAXREF-LD classes “*Daucus carota*” and “*Daucus carota subsp. sativus*.”^7^

Those properties are visible on the property tab of AgroPortal.^8^

The generic properties *ontofcu:hasTaxon ontofcu:hasScientificName* (/ *ontofcu:hasUsage* *ontofcu:hasVernacularName*) can be used to declare any automatic mappings, potentially associated with their score but without validation concern. The generic properties just state that mappings were produced by any automatic method, and they are candidate mappings that need validation. The specific properties will be used to declare valid, cleaned, and precised mappings with their confidence value.

#### 2.1.4. Alignment metadata model SSSOM

Simple standard for Sharing Ontology Mappings (SSSOM) is a recent standard model developed by the biomedical community around OBO Foundry and described by Matentzoglu et al. (2022). It provides a rich set of metadata to describe mappings (individual mappings between a pair of entities) and a mapping set (a set of individual mappings). For this work, we used SSSOM version 0.15 that was released in July 2023.

The main objective of the SSSOM project is to propose a catalog of metadata allowing to have information about the provenance of the mappings, whether they are calculated manually or automatically. The expected impact is an augmented trustworthiness resulting in an increased reuse of mappings by third parties. Meanwhile, there is also a real desire to produce a model that is easy to use. Indeed, the project proposes, on the one hand, the serialization in RDF/OWL for the Semantic Web community and, on the other hand, a TSV format for a larger community which can thus exchange mappings in a simple format yet with rich semantics.

We chose to publish the mapping set in the TSV format as a first step. We opted for the embedded mode where mapping set level metadata are integrated in the mapping TSV file as commented YAML (prefixed with #). The properties used to describe the mapping set are presented in Table 1 while those used to describe each mapping are shown in Table 2. Mandatory properties are marked with an asterisk (*). For each property, we indicate its description as stated in the SSSOM model version 0.15. In Section 3, we present a short analysis of advantages and limits of the SSSOM model.

**Table 1:** SSSOM (V0.15) properties used to describe our mapping set.

**Property**	**Description**	**Example**
mapping_set_id	A globally unique identifier for the mapping set (not each individual mapping). Should be IRI, ideally resolvable.	“https://doi.org/10.57745/LVRFWJ”
creator_id	Identifies the persons or groups responsible for the creation of the mapping. The creator is the agent that put the mapping in its published form, which may be different from the author, which is a person that was actively involved in the assertion of the mapping.	#creator_id: “https://ror.org/01pd2sz18”
creator_label	A string identifying the creator of this mapping.	#creator_label: “Mathématiques, Informatique et Statistique pour l'Environnement et l'Agronomie”
curie_map	A valid curie map that allows the unambiguous interpretation of CURIEs.	#curie_map : #fcu: “http://ontology.inrae.fr/frenchcropusage” #taxref: “http://taxref.mnhn.fr/lod/taxref-ld”
subject_source	URI of ontology source for the subject.	#subject_source: “http://ontology.inrae.fr/frenchcropusage”
subject_source_version	Version IRI or version string of the source of the subject term.	#subject_source_version: “3.3”
object_source	IRI of ontology source for the object. Version IRI preferred.	#object_source: “http://taxref.mnhn.fr/lod/taxref-ld”
object_source_version	Version IRI or version string of the source of the object term.	#object_source_version: “15.2”
license	A url to the license of the mapping. In absence of a license we assume no license.	#license: “https://creativecommons.org/licenses/by/2.0/”

**Table 2:** SSSOM (V0.15) properties used to describe our mappings.

**Property**	**Description**	**Example**
subject_id*	The ID of the subject of the mapping.	fcu:Carottes_fourrageres
subject_label	The label of subject of the mapping.	carotte fourragère
predicate_id*	The ID of the predicate or relation that relates the subject and object of this match.	fcu:def/hasReferenceTaxon
predicate_label	The label of the predicate/relation of the mapping.	Has reference taxon
object_id*	The ID of the object of the mapping.	taxref:taxon/133744
object_label	The label of object of the mapping.	*Daucus carota* subsp.*sativus*
confidence	A score between 0 and 1 to denote the confidence or probability that the match is correct, where 1 denotes total confidence.	1
mapping_justification	A mapping justification is an action (or the written representation of that action) of showing a mapping to be right or reasonable.	semapv: ManualMappingCuration
mapping_cardinality	A string indicating whether this mapping is from a 1:1 (the subject_id maps to a single object_id), 1:n (the subject maps to more than one object_id), n:1, 1:0, 0:1 or n:n group. Note that this is a convenience field that should be derivable from the mapping set.	N:N
subject_type	The type of entity that is being mapped.	skos:Concept
object_type	The type of entity that is being mapped.	owl:Class
author_id	Identifies the persons or groups responsible for asserting the mappings. Recommended to be a (pipe-separated) list of ORCIDs or otherwise identifying URLs, but any identifying string (such as name and affiliation) is permissible.	https://orcid.org/0000-0002-3076-5499
author_label	A string identifying the author of this mapping. In the spirit of provenance, consider to use author_id instead.	Catherine Roussey
reviewer_id	Identifies the persons or groups that reviewed and confirmed the mapping. Recommended to be a (pipe-separated) list of ORCIDs or otherwise identifying URLs, but any identifying string (such as name and affiliation) is permissible.	https://orcid.org/0000-0002-5872-5034
reviewer_label	A string identifying the reviewer of this mapping. In the spirit of provenance, consider to use author_id instead.	Juliette Raphel
mapping_date	The date the mapping was asserted. This is different from the date the mapping was published or compiled in a SSSOM file.	2023-02-03T00:00:00Z
curation_rule_text	The textual representation of curation rule is intended to be used in cases where (1) the creation of a resource is not practical from the perspective of the mapping_provider and (2) as an additional piece of metadata to augment the curation_rule element with a human readable text.	CR_Experts: the experts found the mapping in the Official Catalog of Species and Varieties of Cultivated Crops in France [https://www.geves.fr/catalogue-france/]
comment	Free text field containing either curator notes or text generated by tool providing additional informative information.	Many subspecies of *Daucus carota* can be eaten and used as forage : the most well known is *Daucus carota* subsp. *sativus*

### 2.2. Manual alignment method

Previously, we have tested some automatic alignment methods (Michel et al., 2022) that reused existing reference sources such as EPPO global database (see text footnote ^1^) and the official French catalog of species and varieties of cultivated plants GEVES (see text footnote ^2^). The final automatic method computes a confidence score of the mapping between a taxon from TAXREF-LD and a crop usage from FCU using EPPO database and GEVES catalog. The main problems come from name ambiguity: depending on the source (1) the scientific name may follow or not the botanical nomenclature code used in TAXREF-LD. For example, the scientific name of carrot may be “*Daucus carota*”, “*Daucus carota* L”, or “*Daucus carota* L, 1753”. (2) The vernacular name presents in the source may not match exactly the vernacular name displayed in FCU. For example, the vernacular name of carrot presents in the source may be “carotte sauvage” (“wild carrot”). This name does not appear in FCU. (3) The vernacular name present in the source may not identify precisely a crop usage, that is to say a FCU concept narrower than “*Usages_plantes_cultivees*”. For example, carrot has two crop usages in FCU “carotte potagère” (“vegetable carrot”) and “carotte fourragère” (“folder carrot”). The output of the automatic method should be cleaned by human experts due to the inaccuracy of name comparison.

Thus, we decided to create a new mapping set by asking experts to propose correct and well-known mappings between crop usages, taxa, and their scientific names. All the proposed mappings should have a high confidence value. If any ambiguity existed, the mapping should not be created. First, we provided the experts with guidelines to help them in their decisions. Second, some research tools were proposed to search terms into the two knowledge graphs. Third, three curation rules were written to contextualize the mappings they created and indicate the provenance of the mappings. We focused on specific crops: grapevine, carrot, chicory, and tomato according to the availability of experts.

**Figure 5:**
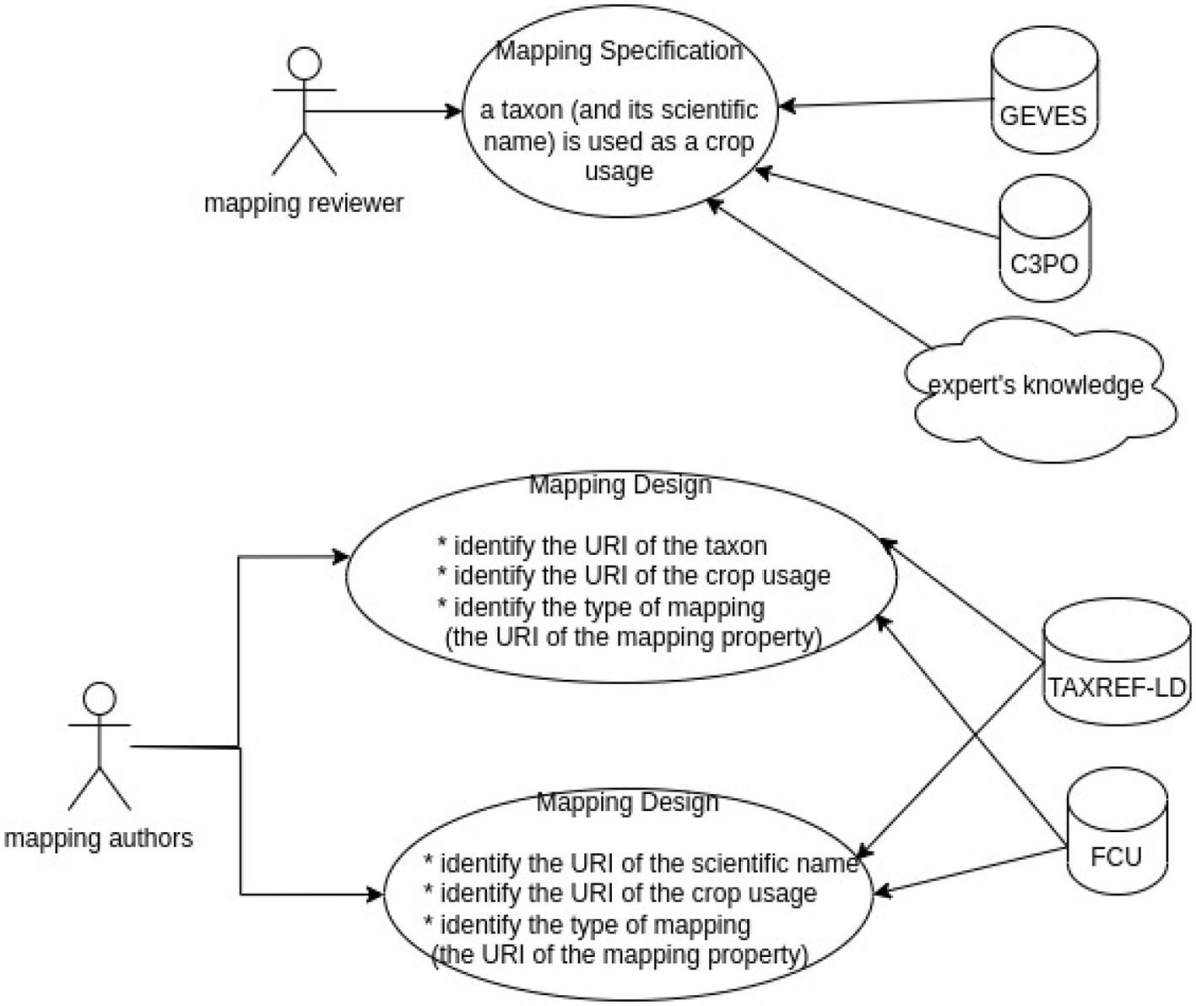
The overview of the method.

As shown in Figure 5, two kind of experts are involved. First, the mapping reviewer proposes some mapping specifications based on its knowledge or other information source. A mapping specification looks like : the taxon “*Daucus carota*” is used as “carotte potagère” (“vegetable carrot”). Second, the reviewer author has to find the correct URI from TAXREF-LD and FCU knowledge graphs to produce the SSSOM mappings following the mapping specifications.

#### 2.2.1. Generic guidelines

We provide the following guidelines to help the experts create their mappings:

1. Only the mappings from FCU crop usages to TAXREF-LD taxa are represented. We focus on crop usages that belong to the branch “*Usages*_*plantes*_*cultivees*” to avoid ambiguity. The goal is to select the most specific crop usage from FCU and align it to some TAXREF-LD taxa using the properties defined in Section 2.1.3.
2. If possible the experts should select a reference information source used to identify the mappings. Each information source is associated with a curation rule to describe the mapping identification method.
3. First, the experts should create a mapping between a crop usage and its reference taxon using the annotation property *ontofcu:hasReferenceTaxon*. We impose that the first reference taxon has the rank species.
4. Second, if a more specific type of taxon, for example, a subspecies or a variety, is well known as the reference taxon of the crop usage, another mapping is created using the annotation property *ontofcu:hasReferenceTaxon*.
5. If the known reference taxon of the crop usage is not available in TAXREF-LD, another type of mapping should be used. A triple based on property *ontofcu:hasGenericTaxon* should be created.
6. Based on the above annotation properties, the mapping between the crop usage and the taxon scientific names are derived. First, a mapping is created between the crop usage and the reference scientific name using the object property *ontofcu:hasReferenceScientificName*. In TAXREF-LD, the reference scientific name of a taxon can be found by following the link *taxref:hasReferenceName*.
7. If the reference source of information (or the experts' knowledge) indicates another scientific name than the one provided in TAXREF-LD as reference name, the experts should try to find it in the synonym names of the taxon and create a new mapping between the crop usage and the scientific name using the object property *ontofcu:hasSynonymeScientificName*.

The name of the experts are indicated in mapping information as mapping reviewer or mapping author. The reviewer is the expert who searched into the reference source the mapping information (or who knew for sure the taxon that fulfills the crop usage). The author is the expert who searched into the knowledge graph the URI entity based on reviewer information. The reviewer information is synthesized into the comment of the mapping (see Table 2).

#### 2.2.2. Search tools

**Figure 6:**
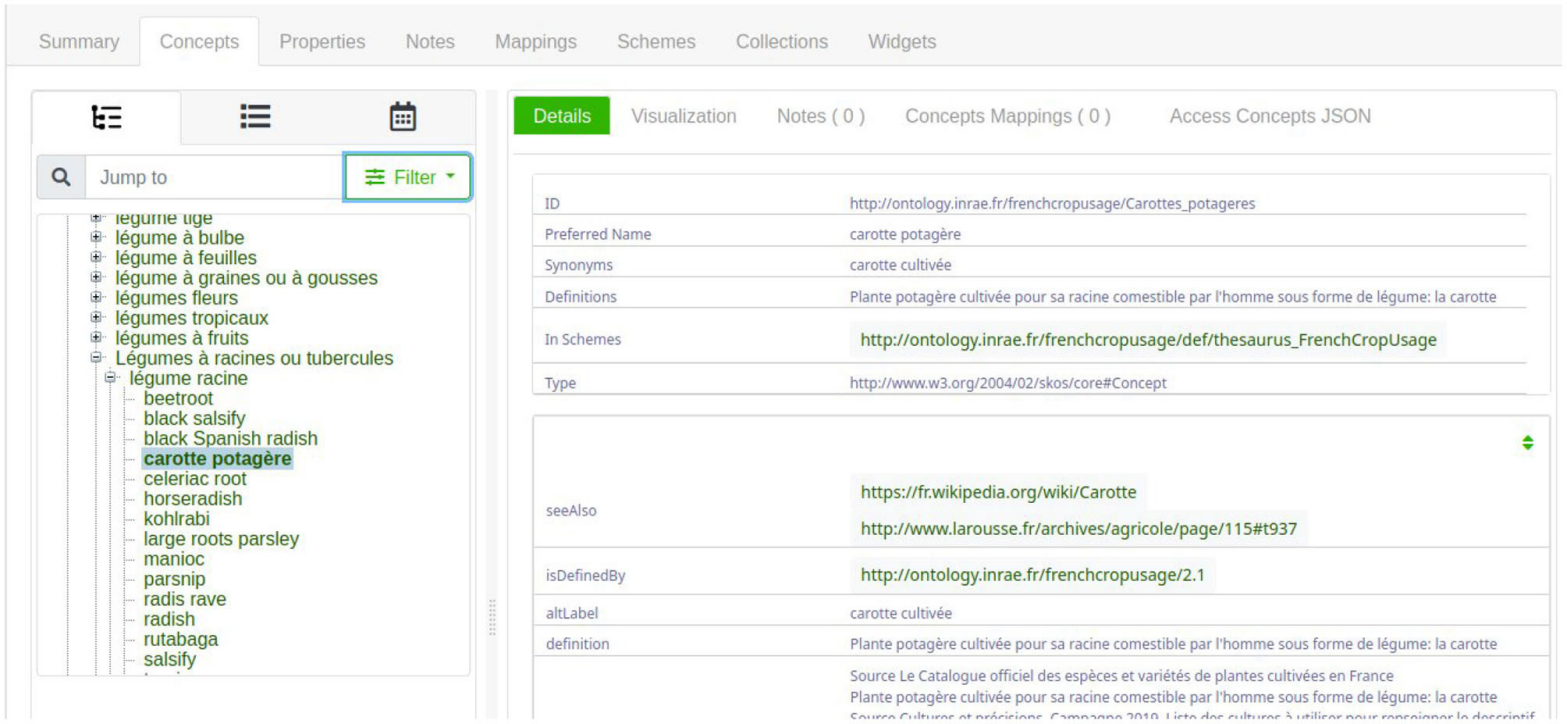
The AgroPortal concept tab interface showing the vegetable carrot crop usage.

To find a crop usage in FCU thesaurus, the following query interfaces are available:

One simple solution is to navigate through the AgroPortal interface concept tab,^9^ to select the most specific instance of *skos:Concept*. Figure 6 shows the hierarchy exploration to find the instance named “*fcu:Carottes_potageres*.” Notably, this interface presents first English labels, and if no English label is provided, the French labels are presented. Thus, Figure 6 presents a mixture of English and French labels.

**Figure 7:**
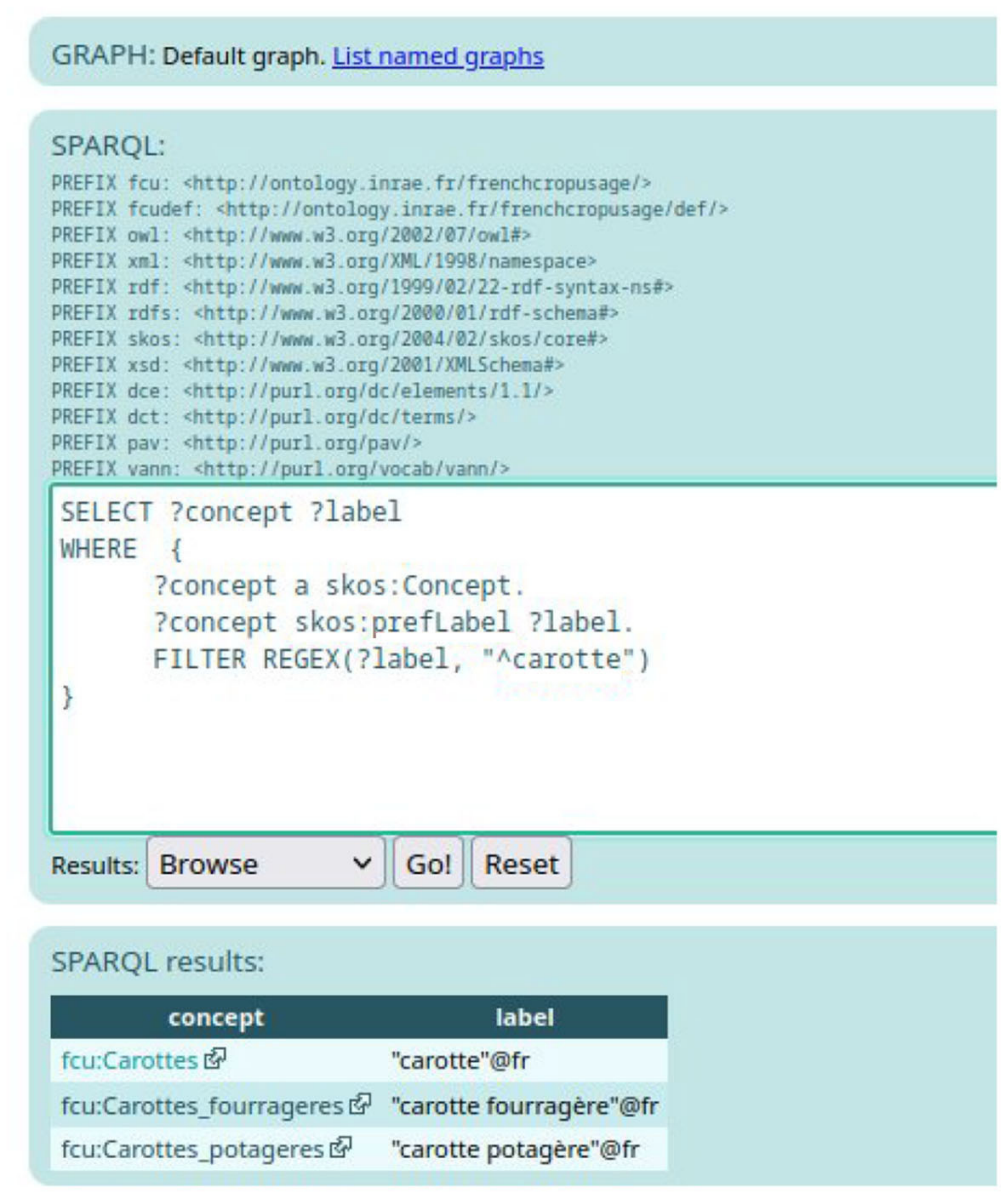
The results of the SPARQL query related to carrot.

To be sure to search French labels, another solution is to query the SPARQL Endpoint.^10^ For example, the query presented in Figure 7 search for an instance of *skos:Concept* that contains the French word “carotte” in their preferred French label.

As shown in Figure 7, there exist three *skos:Concept* instances that have a French preferred label containing the word “carotte.” Remember that the expert should select FCU concept narrower than “*Usages_plantes_cultivees*”. When clicking on one of the results of the SPARQL query displayed on Figure 7, e.g., *fcu:Carrottes_potageres*, the expert accesses the RDF description of the FCU concept, as depicted in Figure 3.

**Figure 8:**
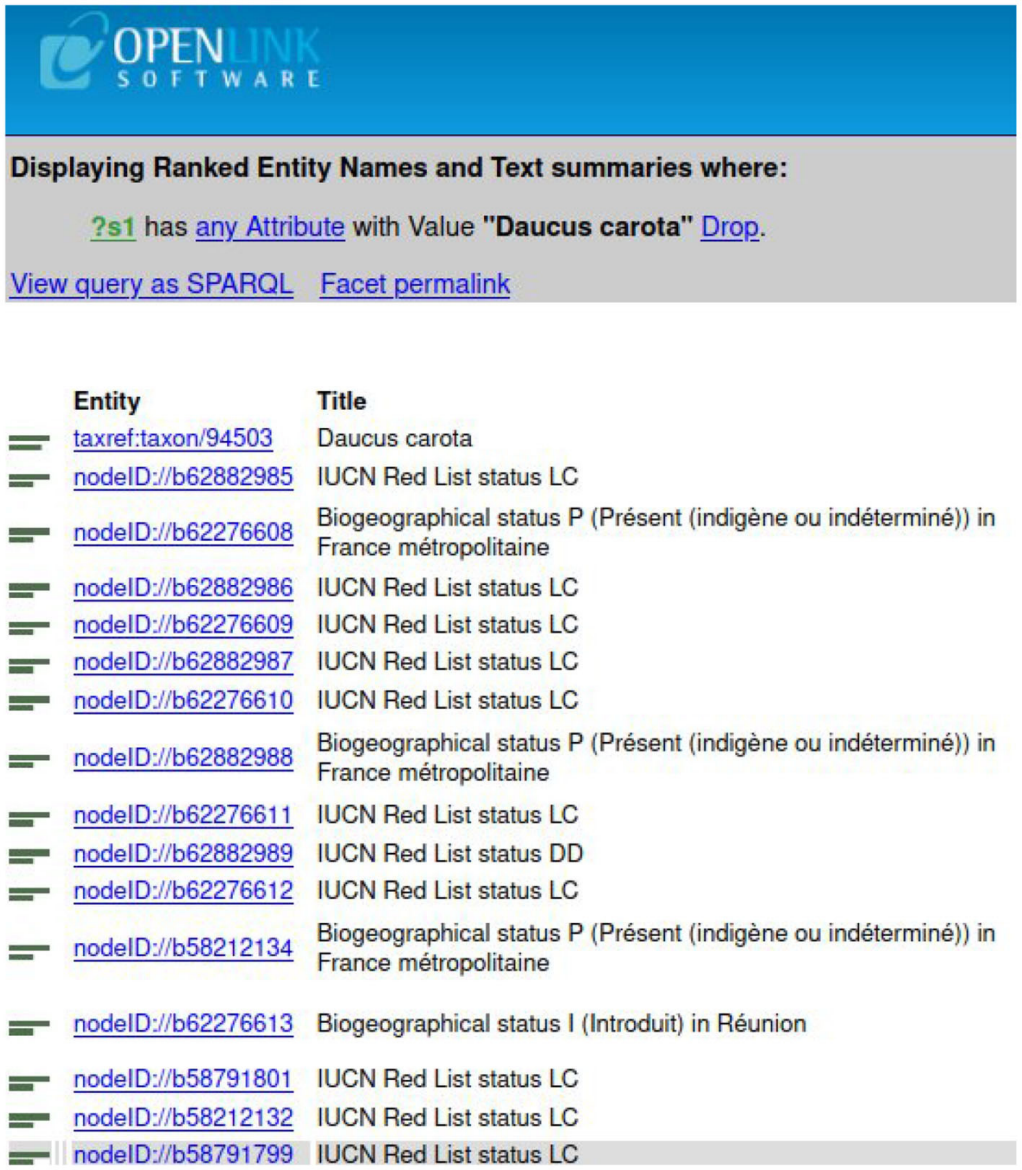
The results of the text query “*Daucus carota*” in TAXREF-LD.

To find a taxon in TAXREF-LD, a text search interface is accessible at http://taxref.i3s.unice.fr/fct/. For example, looking for the text expression “*Daucus carota*” provides the results presented in Figure 8.

**Figure 9:**
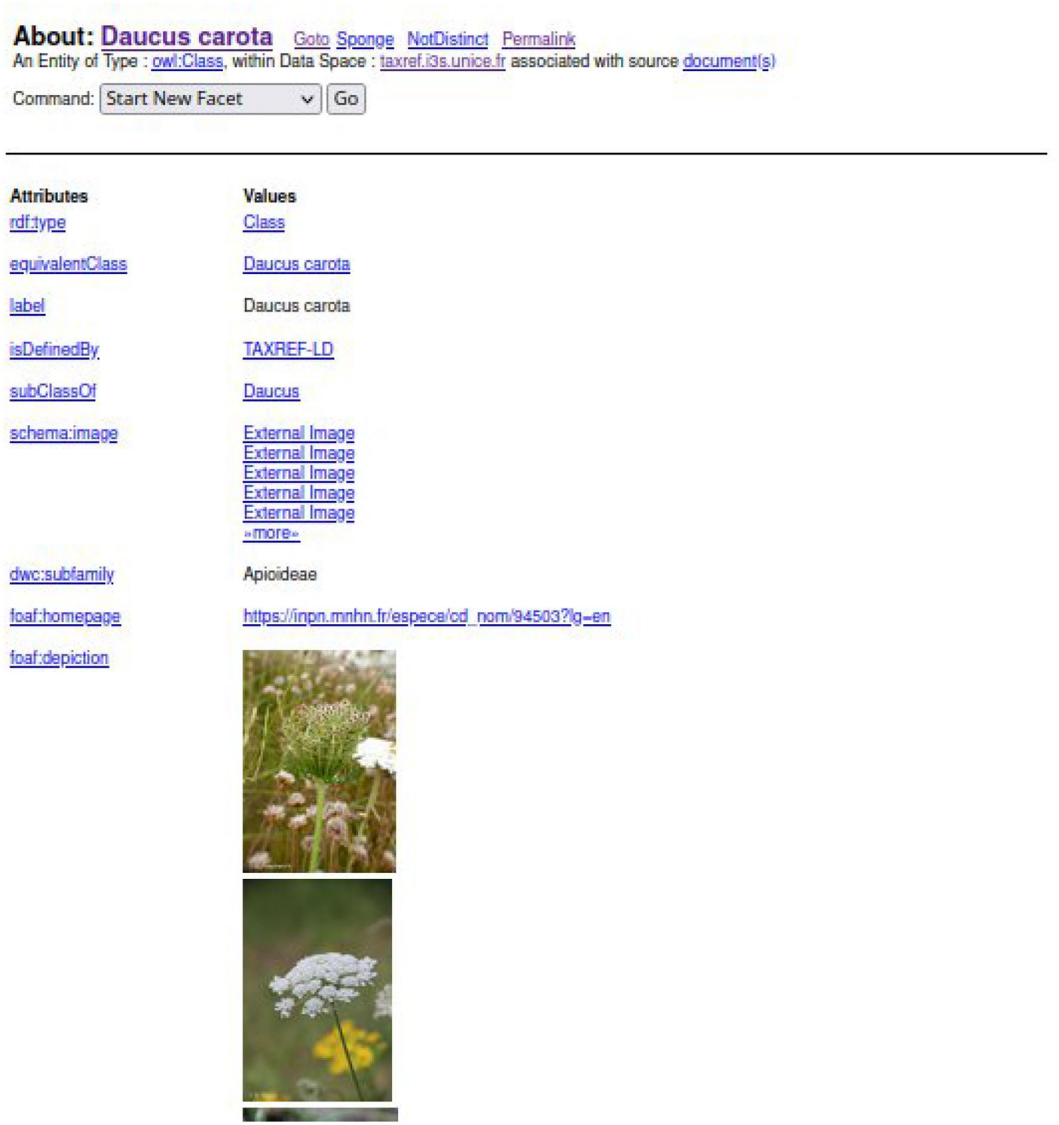
First page of the description of taxon “*Daucus carota*” in TAXREF-LD.

**Figure 10:**
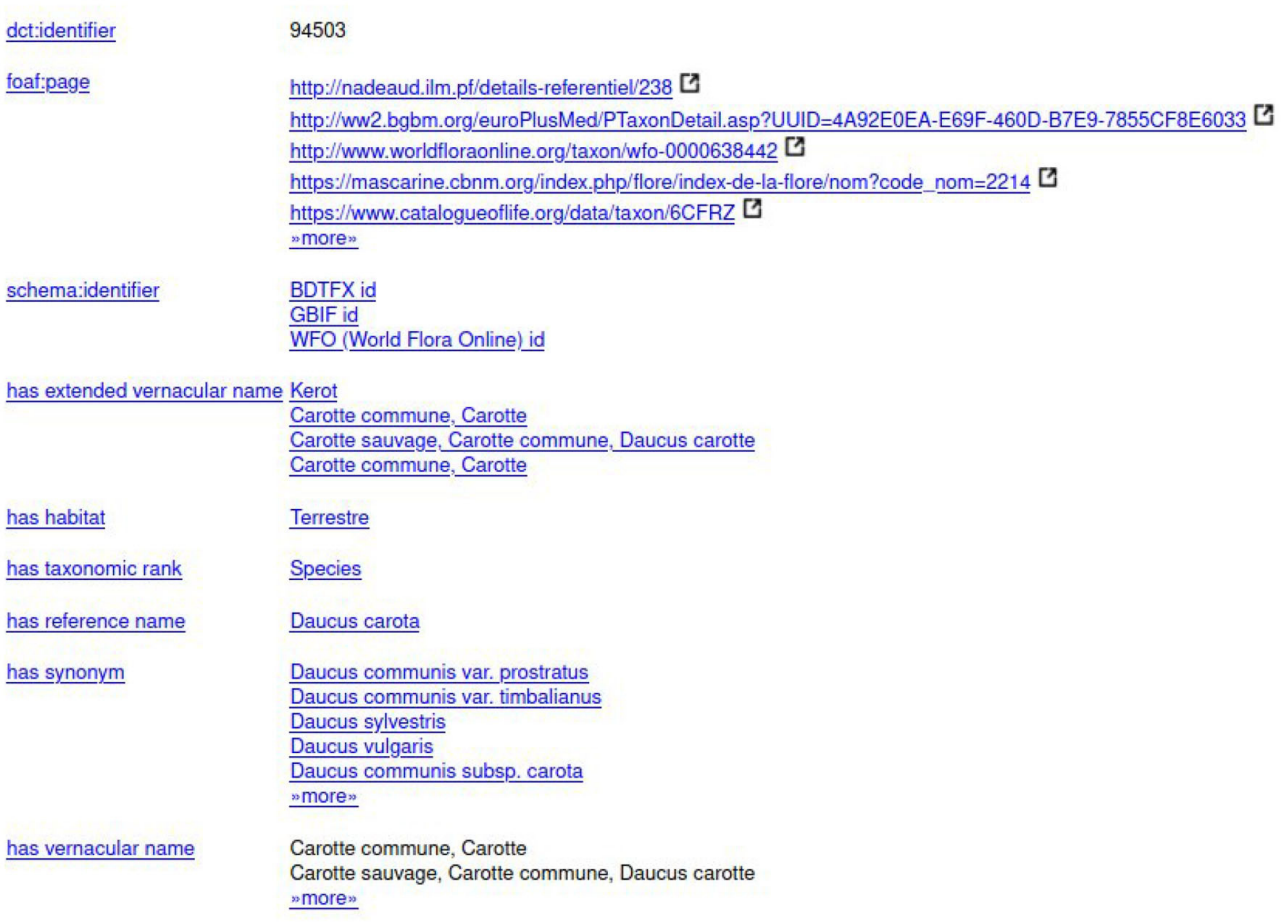
Second page of the description of taxon “*Daucus carota*” in TAXREF-LD.

**Figure 11:**
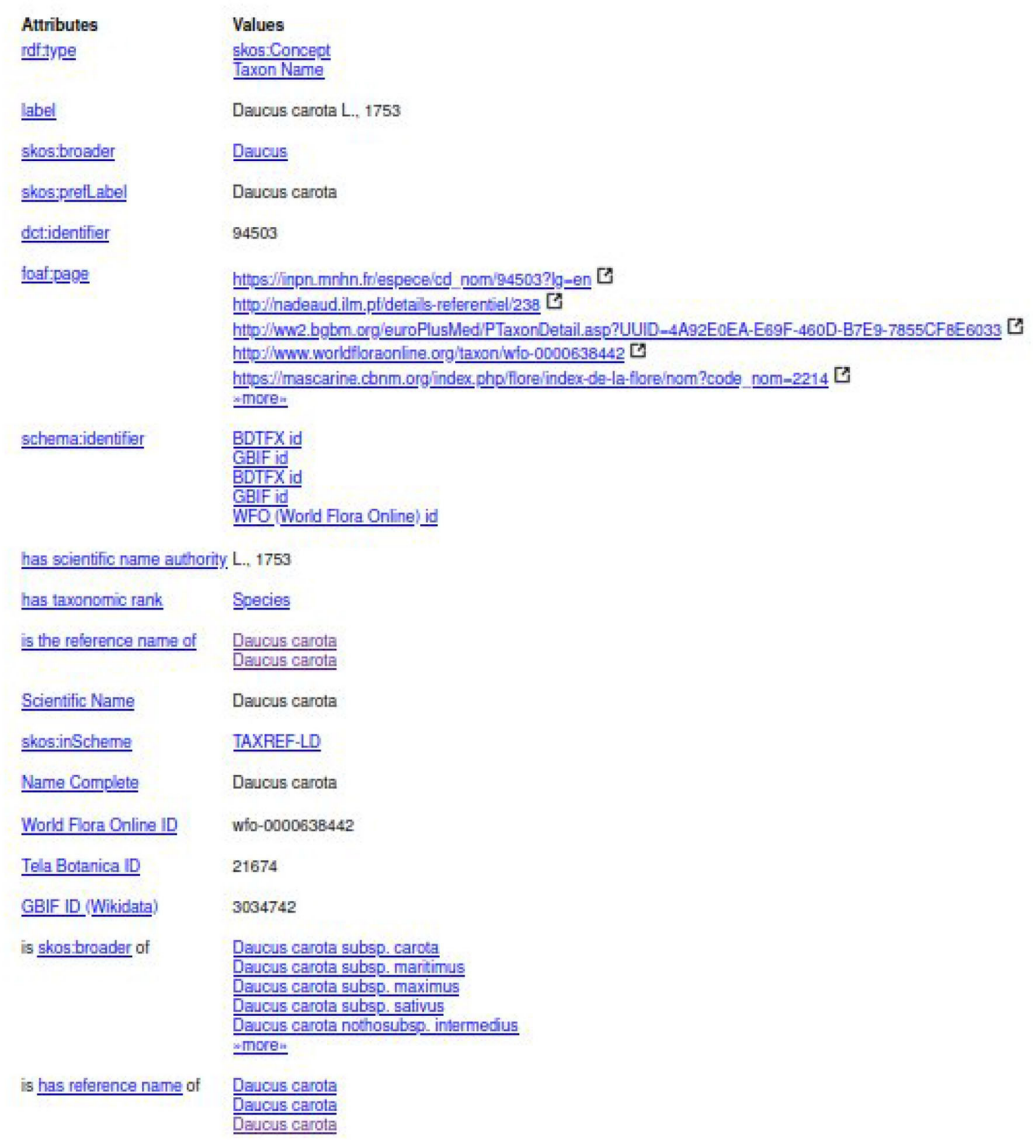
Description of scientific name “*Daucus carota* L., 1753” in TAXREF-LD given as a whole by *rdfs:label* and split into its binomial name “*Daucus carota*” (*skos:prefLabel*) and the authority “L., 1753” (*txrfp:hasAuthority* whose label *has scientific name authority*).

The experts should select the entity of the first line “taxref:taxon/94503.” The prefix “taxref” indicates that the entity belongs to TAXREF-LD. The URL part “taxon” indicates that the entity represents a taxon in TAXREF-LD, that is to say an *OWL class*. Then, the experts click on the Web link to display more information about the entity, as shown in Figures 9, 10. By navigating through the Web interface, the experts follow the property “*has reference name* ” to find its scientific name, as presented in Figure 11.

#### 2.2.3. Curation rules

Three curation rules were created to document our mappings. The goal of curation rules is to prove that the source of information contains the mapping and can be used as mapping justification. Along with the rule, we indicate how to find the relevant elements in each of the mapped resources.

##### 2.2.3.1. CR_Geves

**Figure 12:**
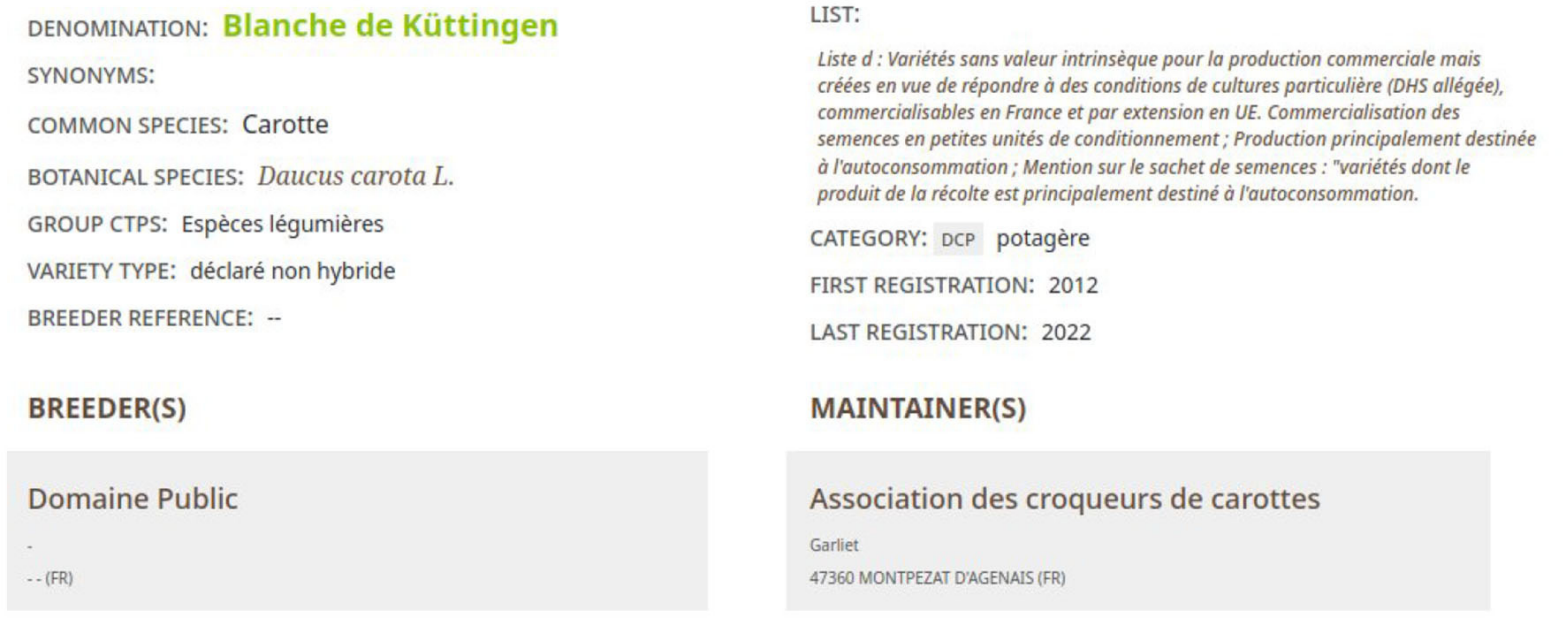
The description of the carrot cultivar “*Blanche de Küttingen*” in the GEVES Catalog.

The *Official Catalog of Species and Varieties of Cultivated Crops in France* from GEVES is a good source of information to find which crop usage is associated with a taxon. As shown in Figure 12, this catalog indicates for each cultivar (seed used by farmers):

- Its vernacular name in the field *Common species*,
- Its crop usage in the field *Category*,
- Its scientific name in the field *Botanical species*.

Notably, most of the time, the taxon rank indicated in this catalog is a species. First, the most specific crop usage was selected in the FCU branch “*Usages*_*plantes*_*cultivees*” by using the information from fields *Common species* and *Category*. Second, the taxon with the scientific name indicated in the field *Botanical species* is searched in TAXREF-LD. If it is possible to find the crop usage and the taxon without ambiguity, the mapping is created and its confidence value is fixed to one.

For example, based on the information provided in Figure 12 about a carrot cultivar, the expert should find in FCU thesaurus the instance of *skos:Concept fcu:Carottes_potageres* (see Figure 7). The expert should also find in TAXREF-LD the instance of *skos:Concept* identified by *taxref:name/94503* (see Figure 11) and the *OWL class* identified by *taxref:taxon/94503* (see Figure 9). Thus, two mappings are created with a confidence value of one:

- One between the *fcu:Carottes_potageres* crop usage and the *taxref:taxon/94503* species taxon using the *ontofcu:hasReferenceTaxon* annotation property.
- One between the crop usage *fcu:Carottes_potageres* and the *taxref:name/94503* reference scientific name using the *ontofcu:hasReferenceScientificName* object property.

##### 2.2.3.2. CR_C3PO_KB

**Figure 13:**
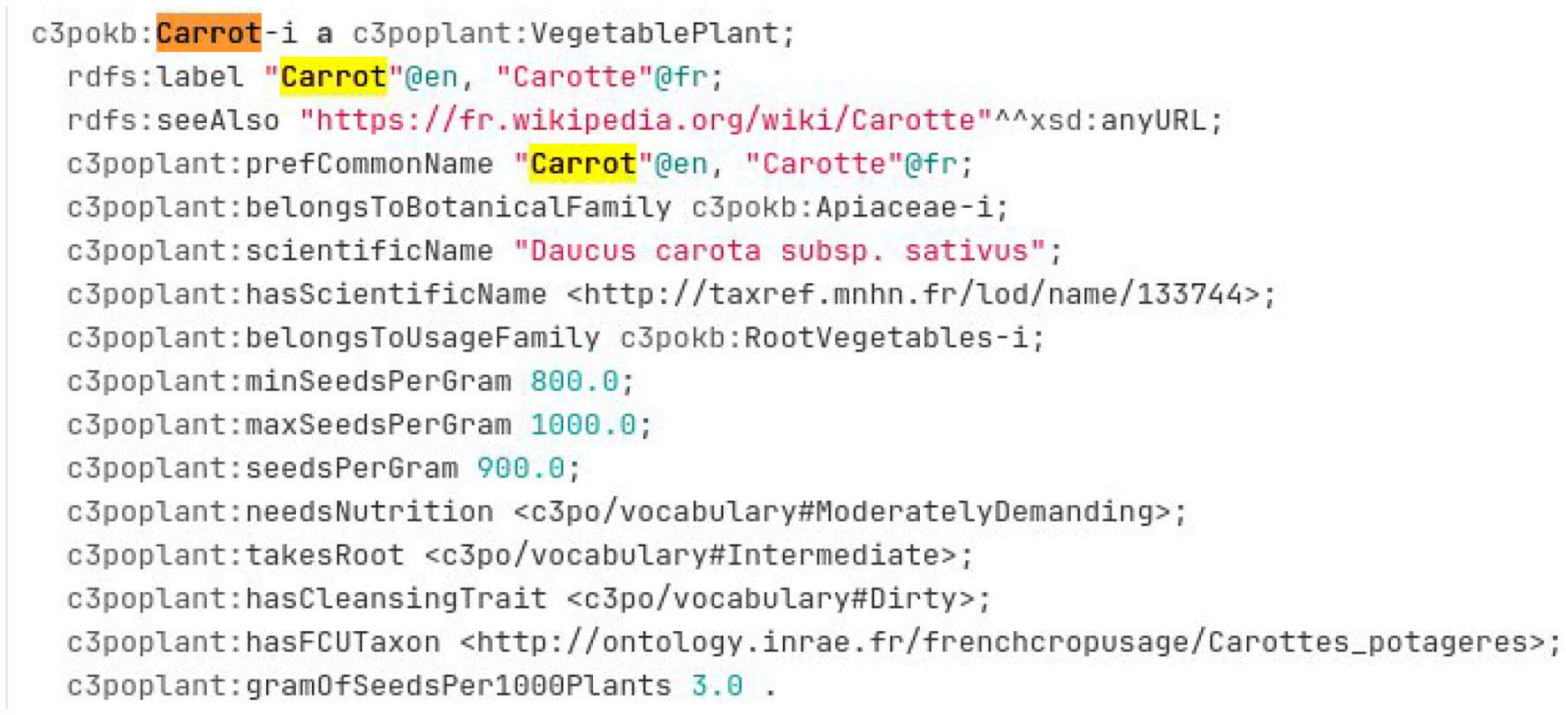
The description of the carrot in the C3PO KB.

The Crop Planning and Production Process Ontology and Knowledge Base (C3PO KB) is a knowledge graph created by the Elzeard Enterprise (Darnala et al., 2021, 2022). This KG is another reference source about vegetable.^11^ The knowledge graph is accessible as several TTL files on a git repository.^12^ To create a mapping between a crop usage and a taxon, the experts should search into the TTL file related to the plant module.^13^ Figure 13 presents an excerpt of this file. The vegetable description contains a crop usage indicated by the property *c3poplant:hasFCUTaxon* and a TAXREF-LD scientific name indicated by the property *c3poplant:hasScientificName*. The experts should find the corresponding taxon by searching in TAXREF-LD Web interface (following the link “*is the reference name of* ”). Based on those information, some new mappings are created with a confidence value of one.

For example, Figure 13 presents the RDF description in TTL format of the instance of *c3poplant:VegetablePlant* related to carrot. Based on this description, the expert can construct two mappings with a confidence value of one as follows:

- One between the *fcu:Carottes_potageres* crop usage and the *taxref:taxon/133744* subspecies taxon using the *ontofcu:hasReferenceTaxon* annotation property.
- One between the crop usage *fcu:Carottes_potageres* and the *taxref:name/133744* reference scientific name using the *ontofcu:hasReferenceScientificName* object property.

##### 2.2.3.3. CR_Experts

The experts can also use their own knowledge to state that a scientific name is linked to a given crop usage. In this case, the taxon name is searched in TAXREF-LD. If the scientific name is retrieved and this name is the reference name for the taxon, two mappings are created as follows:

- One between the crop usage and the taxon using the *ontofcu:hasReferenceTaxon* annotation property.
- One between the crop usage and the scientific name using the *ontofcu:hasReferenceScientificName* object property.

If the scientific name is retrieved but the name is not the reference name for the taxon but a synonym name, three mappings are created as follows:

- One between the crop usage and the taxon using the *ontofcu:hasReferenceTaxon* annotation property.
- One between the crop usage and the reference scientific name using the *ontofcu:hasReferenceScientificName* object property.
- One between the crop usage and the synonym name using the *ontofcu:hasSynonymousScientificName* object property.

If the scientific name is not retrieved (for example, “*Cichorium intybus var. foliosum Hegi f. cylindricum*” is the scientific name of a form taxon), but the name of a parent taxon can be retrieved (“*Cichorium intybus var. foliosum Hegi* ” is the scientific name of the variety taxon), the experts should restart the process by looking for the reference scientific name of the parent taxon. This implies that the properties used will be *ontofcu:hasGenericTaxon* and *ontofcu:hasGenericScientificName*.

The confidence value all these mappings is fixed to one.

## 3. Analysis

### 3.1. Alignment challenges

Although the taxonomies and lists of cultivated plants (or crop usages) refer to living organisms, their alignment raises several challenges that require manual curation in practice.

A taxonomy is a hierarchical structure that presents a set of hypotheses uttered by taxonomists on taxa and their relationships. The names of taxa are regulated by written conventions recorded in nomenclature codes. The current code in force for plants is the *International Code of Nomenclature for algae, fungi, and plants*, also known as the *Shenzhen Code* (Zhang et al., 2013). By their very nature, taxonomies change over time as the scientific consensus between taxonomists evolves. Taxa may merge, split, spawn, or change taxonomic rank (e.g., from *species* to *subspecies*). Nomenclature codes acknowledge this fact and were designed to stabilize taxon names as much as possible, given the versatility of taxonomies.

At a given time, a taxon has a single valid name and possibly a list of synonyms that have spawned from previous studies. Changes in the nomenclature are published on an *ad hoc* basis in the scientific literature. However, taxonomies and lists of cultivated plants are updated at a different pace. As a consequence, different references might not be completely up-to-date and may exhibit discrepancies on specific taxa. For instance, a list of cultivated plants may use a scientific name which is no longer valid or whose taxon has changed parent taxon or rank.

Additionally, nomenclature codes apply unambiguously to the species and subspecies taxon ranks but not to the lower ranks (e.g., *variety* or *cultivar*). On the other hand, the naming of cultivated plants is not regulated in any way, and a cultivated plant name is not even required to refer to a scientific taxon.

Furthermore, due to their complexity, nomenclature codes are not always strictly observed. For instance, the *Official Catalog of Species and Varieties of Cultivated Crops in France* gives the authority without the date (e.g., “L.” instead of “L. 1758”). Lists of cultivated plants are often built by aggregating several primary sources. However, these resources seldom cite primary sources, hindering the assessment of the confidence of their content.

Finally, vernacular names used to denote cultivated plants are sometimes specific to a given region, making alignment locale-dependent.

There are more technical difficulties related to modeling choices for representing taxa, names, and cultivated plants. For example, TAXREF-LD (Michel et al., 2017) strictly separates taxonomy and nomenclature. Other resources do distinguish taxonomy and nomenclature, representing both taxa and their names at the same level. Some classifications represent only scientific names, such as the Catalog of Life (Hobern et al., 2021). The lists of cultivated plants often retain only a scientific name instead of a taxon, a name which may no longer be valid.

These design and modeling variations raise recurring questions on the type of objects to be aligned: do we align two taxa, or a taxon and a name, or a cultivated plant and a taxon, etc.?

Facing these challenges, we have proposed to define new mapping properties to represent the link between a crop usage, a taxon, and its scientific name.

### 3.2. SSSOM model analysis

SSSOM is a Simple Standard for Sharing Ontological Mappings which provides means to represent rich metadata for mappings and mapping sets. This standard representation has several advantages as follows:

- Entities of different nature can be mapped, for example, an instance of *owl:Class* with an instance of *skos:Concept*.
- SSSOM does not impose the use of specific mapping properties such as *owl:equivalentClass*, the *oboInOwl hasDbXref*, or the well-known SKOS mapping properties, e.g., *skos:exactMatch*. In the context of this study, we were, thus, able to define the mapping properties relevant to our application (see Section 2.1.3).
- A set of mappings can become a dataset to be stored and shared independently of the aligned resources. Provenance metadata at dataset level allow to document the context in which the mappings are valid, for example, for the needs of an application. It is also possible to publish successive versions of a mapping set in a transparent way.
- Mappings can be described as first class objects and be referenced individually with an identifier (URI). This allows to declare equivalences between mappings coming from various sources to build aggregated mappings.
- Authors and reviewers of the mappings can be credited for their study as SSSOM recommends the use of ORCIDs with the author_id and reviewer_id properties.

The SSSOM project provides a rich documentation with many examples which facilitates the study. In addition, the SSSOM community is very active in working on improving and extending the model based on feedback from use cases issued from varied communities. This study of publishing mappings led us to contribute to the discussions taking place on the SSSOM github repository.^14^ We expressed our need to document the rules that led us to assess a given mapping and discussed with the SSSOM authors on how to represent and expose these rules. As this new feature was plebiscited by several users, two new properties *sssom:curation_rule* and *sssom:curation_rule_text* were recently added to the SSSOM model. The rule can be represented either directly in the mapping set (using sssom:curation_rule_text) or be published as a RDF resource and be referenced with its URI in the mapping set (using sssom:curation_rule). As we work in TSV, we decided to give a short version of each curation rule in the TSV file of the mapping set, to refer to this article for a much finer description of our curation rules 2.2.3.

SSSOM, however, still shows some limitations or elements that are not fully mature as follows:

- Complex mappings: In our case, a crop usage may be fulfilled by a combination of taxa; for instance in viticulture, a vine plant can be composed of a rootstock and a graft. The rootstock is the buried part of the vine and serves as a support for the graft. Here, we may want to align *crop A* with a combination of a *taxon B* (rootstock) and a *taxon C* (graft) and indicate their respective roles. The future extension of SSSOM to handle complex mappings may include a property to assert the role of each component of a combination. If so, our RDF vocabulary would be extended to provide the types of roles in our specific context.
- Negative mappings: the open world assumption holds in Semantic Web models. This means that the absence of mapping does not mean that this does not exist or is incorrect. Moreover, we want to declare that some mappings are false or irrelevant. The SSSOM community investigated two solutions: adding a modifier column or creating negative mapping properties. As we developed our own mapping vocabulary, we decided to create some specific mapping properties to indicate that a mapping should not exist between FCU and TAXREF-LD: *ontofcu:hasInvalidTaxon* / *ontofcu:hasInvalidUsage* / *ontofcu:hasInvalidScientificName* / *hasInvalidVernacularName*.
- Confidence is defined in SSSOM as “A score between 0 and 1 to denote the confidence or probability that the match is correct, where 1 denotes total confidence.” We are wandering if this property is relevant in the case of manual alignment. Concretely, the experts working on this mapping set faced two difficulties in filling out this field. First, it was difficult to fix a confidence value when they were not completely sure about a mapping, or even worse, when they disagreed. For this reason, and also because the SSSOM property lacks some clarity and is actually under discussion, we agreed on publishing only mappings with a confidence value equal to one.
- Cardinality: as recommended in the guidelines, this property should be automatically filled in. It took a long time to compute the values and would be difficult to maintain when more mappings are added to the mapping set.

Overall, SSSOM is a rising metadata standard for sharing, analyzing, and integrating mappings. It covers our needs pretty well. The SSSOM project also offers a forum to discuss solutions with experts and practitioners from various domains.

## 4. Conclusion and perspectives

This article describes our work on the alignment of two complementary knowledge graphs useful in agriculture: the crop usage defined in the thesaurus of cultivated plants in France named French Crop Usage (FCU) and the taxa and associated scientific names defined in the French national taxonomic repository TAXREF for fauna, flora, and fungi. Due to the fact that automatic alignment methods provide poor results, a group of agricultural experts has produced a set of valid mappings between crop usages, taxa, and associated scientific names. To do so, a new RDF vocabulary of mapping properties was defined to align those plant descriptions. The metadata for the mappings and the mapping set are encoded with the Simple Standard for Sharing Ontological Mappings (SSSOM), a new model which offers means to report on the mapping provenance. To help the mapping creation, we provided some guidelines and tools to the experts. The produced mappings are available for download in Recherche Data Gouv, the federated national platform for research data in France.

Those mappings can be viewed as a first effort to test SSSOM Model using the TSV format. The mappings are manual and simple ones with high confidence value. Thus, they represent valid and consolidate mappings. We would like to enrich this mapping set by taking into account the whole Catalog of GEVES and C3PO KB. Both are evaluated as good source of information by our experts. We also plan to exploit these valid mappings to evaluate automatic alignment methods. The difficulty will be to manage the evolution of FCU and TAXREF-LD and keep up to date the mappings between those graphs. We would like to detect automatically obsolete mappings. This study is a first step to test the description of mappings produced by experts. We hope that it can help other use cases of mapping between complementary description defining different points of view on the same objects of study. We also hope that the provision of curated mappings will allow testing of new automatic methods capable of working with scientific names and vernacular names.

## Data availability statement

The datasets presented in this study can be found in online repositories. TAXREF-LD: The version 15.2 of TAXREF-LD graph used for this study can be found in the AgroPortal repository https://agroportal.lirmm.fr/ontologies/TAXREF-LD. The github repository is https://github.com/frmichel/taxref-ld. The SPARQL EndPoint is https://taxref.mnhn.fr/sparql. FCU: The version 3.3 of the FCU thesaurus used for this study can be found in the AgroPortal repository: https://agroportal.lirmm.fr/ontologies/CROPUSAGE. The gitlab repository is https://gitlab.irstea.fr/copain/frenchcropusage. The SPARQL EndPoint is http://ontology.inrae.fr/frenchcropusage/sparql. SSSOM: The github repository is https://github.com/mapping-commons/sssom C3PO KB: The version 1.0 of the C3PO KB can be found in the gitlab repository https://gitlab.com/serre-des-savoirs/c3po-kb. The associated ontology can be found on the AgroPortal repository https://agroportal.lirmm.fr/ontologies/C3PO/?p=summary mapping set FCU TAXREF-LD. The mapping set between FCU and TAXREF-LD generated for this study can be found in the Research Data Gouv repository https://doi.org/10.57745/LVRFWJ the CSV file version presented in the article is available in article/Supplementary material.

## Author contributions

SA and SB: SSSOM documentation. CF and FM: conceptualization and design of TAXREF-LD knowledge graph and associated research tool. CR and SB: conceptualization and design of FCU knowledge graph and associated research tool. FA and JR: conceptualization and design of C3PO knowledge graph. CR, FA, and JR: guidelines definition and test. CR and SA: writing—original draft preparation. CR, SA, CF, FM, and RB: writing—review and editing. CR: supervision. All authors contributed to the article and approved the submitted version.

## Footnotes

^1^ https://gd.eppo.int/

^2^Catalogue officiel des espèces et variétés de plantes cultivées en France accessible at https://www.geves.fr/catalogue/.

^3^ https://doi.org/10.57745/LVRFWJ

^4^ https://agroportal.lirmm.fr/ontologies/TAXREF-LD

^5^ https://agroportal.lirmm.fr/ontologies/CROPUSAGE/?p=summary

^6^ https://www.geves.fr/catalogue-france/

^7^The UML based CHOWLK language is used to present the knowledge graph. More information available on https://chowlk.linkeddata.es/notation.html.

^8^ https://agroportal.lirmm.fr/ontologies/CROPUSAGE/?p=properties

^9^ https://agroportal.lirmm.fr/ontologies/CROPUSAGE/?p=classes

^10^ http://ontology.inrae.fr/frenchcropusage/sparql

^11^The ontology is accessible on AgroPortal repository https://agroportal.lirmm.fr/ontologies/C3PO/?p=summary.

^12^ https://gitlab.com/serre-des-savoirs/c3po-kb

^13^The file is untitled “cultivatedplants-infos-result-triples.ttl” and is accessible at https://gitlab.com/serre-des-savoirs/c3po-kb/-/blob/main/plants/rdf-generated%20triples/cultivatedplants-infos-result-triples.ttl.

^14^ https://github.com/mapping-commons/sssom/issues

## Supplementary material

SUPPLEMENTARY DATA SHEET 1 SSSOM files that contains 63 mappings between vegetables or grapevine crop usage and their biological taxa.

## References

[B1] Darnala B. Amardeilh F. Roussey C. Jonquet C. (2021). “Crop Planning and Production Process Ontology (C3PO), a new model to assist diversified crop production,” in IFOW 2021 - Integrated Food Ontology Workshop @ 12th International Conference on Biomedical Ontologies (ICBO) (Bolzano, Italy).

[B2] Darnala B. Amardeilh F. Roussey C. Todorov K. Jonquet C. (2022). “Ontological representation of cultivated plants: linking botanical and agricultural usages,” in MK 2022 - 1st Workshop on Modular Knowledge @ ESWC 2022 of CEUR Workshop Proceedings, eds L. Bozzato, V. A. Carrier, T. Hahmann, and A. Zimmermann (Hersonissos, Greece), 165–173.

[B3] Gargominy O. Tercerie S. Régnier C. Ramage T. Dupont P. Daszkiewicz P. . (2021). TAXREF v15, référentiel taxonomique pour la France: méthodologie, mise en uvre et diffusion. Technical report.

[B4] Hobern D. Barik S.K. Christidis L. Garnett S. T. Kirk P. Orrell T. M. . (2021). Towards a global list of accepted species VI: The Catalogue of Life checklist. Org. Divers. Evol. 21, 677–690. 10.1007/s13127-021-00516-w

[B5] Matentzoglu N. Balhoff J. P. Bello S. M. Bizon C. Brush M. Callahan T. J. . (2022). A simple standard for sharing ontological mappings (SSSOM). Database 2022, baac035. 10.1093/database/baac035 35616100 PMC9216545

[B6] Michel F. Amardeilh F. Bossy R. Faron C. Roussey C. Noûs C. (2022). “Alignement entre sources : cas d'usage des plantes cultivées,” in Journées francophones d'Ingénierie des Connaissances (Saint-Étienne, France).

[B7] Michel F. Gargominy O. Tercerie S. Faron-Zucker C. (2017). “A model to represent nomenclatural and taxonomic information as linked data. Application to the french taxonomic register, TAXREF,” in Proceedings of the ISWC2017 workshop on Semantics for Biodiversity (S4BioDiv) (Vienna, Austria: CEUR Workshop Proceedings). Available online at: http://ceur-ws.org/Vol-1933/paper-3.pdf (accessed September 01, 2023).

[B8] Miles A. Bechhofer S. (2009). SKOS Simple Knowledge Organization System Reference. W3C Recommendation. World Wide Web Consortium, United States.

[B9] Zhang N. Rossman A. Seifert K. Bennett J. Cai Y. Hillman B. . (2013). Impacts of the international code of nomenclature for algae, fungi, and plants (melbourne code) on the scientific names of plant pathogenic fungi. APS Journal, Available online at: https://www.apsnet.org/edcenter/apsnetfeatures/Pages/Melbourne.aspx (accessed September 01, 2023).

